# Accelerating the Development of Measles and Rubella Microarray Patches to Eliminate Measles and Rubella: Recent Progress, Remaining Challenges

**DOI:** 10.3389/fpubh.2022.809675

**Published:** 2022-03-02

**Authors:** Mateusz Hasso-Agopsowicz, Natasha Crowcroft, Robin Biellik, Christopher J. Gregory, Marion Menozzi-Arnaud, Jean-Pierre Amorij, Philippe-Alexandre Gilbert, Kristen Earle, Collrane Frivold, Courtney Jarrahian, Mercy Mvundura, Jessica J. Mistilis, David N. Durrheim, Birgitte Giersing

**Affiliations:** ^1^Immunization, Vaccines and Biologicals, World Health Organization, Geneva, Switzerland; ^2^Independent, La Rippe, Switzerland; ^3^Immunization Unit, Programme Division, United Nations Children's Fund (UNICEF), New York, NY, United States; ^4^Gavi, The Vaccine Alliance, Geneva, Switzerland; ^5^Supply Division, Vaccine Centre, UNICEF, Copenhagen, Denmark; ^6^Vaccine Development and Surveillance, Bill and Melinda Gates Foundation, Seattle, WA, United States; ^7^Medical Devices and Health Technologies, PATH, Seattle, WA, United States; ^8^Medicine and Public Health, University of Newcastle, Callaghan, NSW, Australia

**Keywords:** measles, vaccine, rubella, microarray patches, innovation

## Abstract

Measles and rubella microarray patches (MR-MAPs) are critical in achieving measles and rubella eradication, a goal highly unlikely to meet with current vaccines presentations. With low commercial incentive to MAP developers, limited and uncertain funding, the need for investment in a novel manufacturing facility, and remaining questions about the source of antigen, product demand, and regulatory pathway, MR-MAPs are unlikely to be prequalified by WHO and ready for use before 2033. This article describes the current progress of MR-MAPs, highlights challenges and opportunities pertinent to MR-MAPs manufacturing, regulatory approval, creating demand, and timelines to licensure. It also describes activities that are being undertaken by multiple partners to incentivise investment in and accelerate the development of MR-MAPs.

## Section 1: MR-MAPs are Critical Tools for Achieving the Eradication of Measles and Rubella

Measles vaccination is responsible for the highest number of vaccine-preventable deaths averted in children and the greatest return on investment (1, 2). Despite this impact, lack of measles vaccination resulted in more than 200,000 deaths globally in 2019 (3). More cases and measles deaths are expected as the COVID-19 pandemic is having a detrimental impact on vaccine coverage (4). Although measles and rubella (MR) elimination was achieved in the Region of the Americas using currently available vaccines and World Health Organization (WHO) -recommended delivery strategies, gaps in population measles-rubella immunity profiles in many lower- and middle-income countries (LMICs) continue to sustain a costly cycle of sporadic outbreaks and public health responses. Inequities in measles immunization delivery in many LMICs have left behind a relatively steady number of MR “zero-dose” and incompletely vaccinated children who live disproportionately in under-served communities particularly in remote rural, fragile or conflict-affected areas (5).

Current MR vaccines supplied to LMICs are packaged in 5- or 10-dose vials, stored and transported at 2–8°C, require reconstitution with bundled diluent, are heat- and light-labile, delivered using needle and syringe (N&S) by skilled healthcare workers, must be discarded 6 h after reconstitution, and generate significant amounts of medical waste (6). These characteristics create opportunities for logistical failures and programmatic errors related to storage, reconstitution or administration and make it challenging to reliably deliver MR vaccine in areas with few primary healthcare and waste disposal facilities, limited cold chain and supply chain systems, few available skilled healthcare workers, difficult transportation and extreme climatic and environmental conditions. They also force healthcare workers to choose between vaccinating every eligible child brought to a vaccination session or wasting vaccine due to opening multidose vials when there are fewer children present than doses in the vial.

Innovative vaccine presentations, coupled with strategies tailored to their delivery, could help to address some of the challenges associated with N&S delivery and raise immunity profiles to levels required to achieve and sustain MR elimination globally (7). Microarray patches (MAPs) consist of hundreds to thousands of micro projections that deliver a dose of MR vaccine into the dermis. Their characteristics offer potential programmatic advantages over N&S vaccine delivery: ready-to-deploy without reconstitution; single-dose presentation reducing wastage; potential to be administered by community health workers with limited training; relatively painless, sharps-free delivery for increased acceptability; and reduced sharps waste for disposal. These characteristics together with potential thermostability extend the possibility for vaccination beyond the end of the cold chain, allowing for delivery in the most remote and challenging settings, such as conflict and humanitarian crisis regions and communities of refugees or displaced persons. MR-MAPs should overcome many of the logistical obstacles currently exacerbating inequitable MR coverage and hindering MR elimination, reduce the number of zero-dose children, and facilitate the integrated delivery of health services.

Despite their highly anticipated benefits, the first two MR-MAP candidates have only recently entered phase I clinical trials, funded by the Bill and Melinda Gates Foundation (BMGF). The development of an MR-MAP product with WHO prequalification is a pre-requisite for Gavi, the Vaccine Alliance (Gavi) financing, and subsequent procurement by UNICEF for LMIC markets. However, to be WHO prequalified (PQ'd) before 2030, significant investment in the manufacturing infrastructure for MR-MAPs is required at-risk, i.e., in parallel to the clinical development.

Insufficient understanding of country needs and preferences as well as unclear health and economic value of MR-MAPs to countries can result in a lack of clear demand and commercial incentive to vaccine manufacturers, MAP developers and funders, who will be reluctant to invest in MR-MAP product development. This article highlights key barriers and drivers of MR-MAP product development; summarizes activities to identify and articulate the market and value of MR-MAPs to countries, funders, and manufacturers; and provides expert recommendations for critical activities that are needed to expedite the development of and access to MR-MAPs.

## Section 2: Overview of the Clinical Development of MR-MAPs

Two MR-MAP candidates, one with vaccine-coated and one with dissolving microneedles, are currently in early-stage clinical development. The attributes of both candidates are presented in Table 1. These MAP platforms have already demonstrated safety and clinical proof-of-concept (PoC) with seasonal influenza vaccine, eliciting equivalent immune responses to intramuscular administration (9, 10). The coated influenza MAP has also demonstrated dose sparing, with 1/6 of a dose eliciting similar immune responses to a full intramuscular dose for one antigen (9). The dissolving influenza MAP has demonstrated at least 5-fold dose sparing in preclinical studies and human trials (10). The reactogenicity and acceptability of the dissolving MAP format has been assessed as a placebo-MAP in infants (NCT03207763) and a similar study is planned for the coated placebo-MAP in 2022.

**Table 1:** Summary of attributes and considerations for two MR-MAP products and their current alignment with MR-MAP Target Product Profile (TPP).

**Attribute or consideration**	**Target Product Profile (minimally acceptable target)**	**Vaxxas (www.vaxxas.com/)**	**Micron Biomedical (micronbiomedical.com/)**
MAP presentation	A single dose coated or dissolving MR vaccine delivery device.	Single dose, high density (HD) MAP with solid micro-projections coated with MR vaccine in a primary container that is also the applicator.	Single dose dissolving MR vaccine delivery device.
Antigen supply for clinical phase one	MR antigens should be prequalified by WHO.	SIIPL (WHO prequalified)	SIIPL (WHO prequalified)
MR immunogenicity data from animal models	Not applicable	Yes	Yes (8)
Wear time	Wear time up to 5 min, under observation, before removal of MAP by healthcare workers, trained lay health worker or caregiver.	Anticipated wear time of ≤ 1 min.	5 min in ongoing Phase $\frac{1}{2}$ trial. Target of <1 min and possibly <10 s in commercial product.
Demonstration of temperature stability	Vaccine potency stability profiles should be superior to current MR vaccine stability, i.e., vaccine vial monitor 14 when stored at 2–8°C (24 months), and must be amenable to controlled temperature chain, i.e., a single excursion for at least 3 days at 40°C.	<1 log loss in potency after 9 months at 25°C (60% relative humidity), or 14 days at 37°C (60% relative humidity). for M and R vaccines. Stability studies are ongoing.	At least 6 months at 25°C and 60% relative humidity for MR for consistency with ongoing stability studies.
Plans for clinical trials	Not applicable	Phase I, randomized, partially blind, age 18–50, in (Australia). The intervention is MR delivered by HD-MAP at two different dose levels and the objectives are safety and tolerability, and immunogenicity.	Phase I/II study, randomized, double-blind, age de-escalation in the Gambia. The intervention is MR delivered by MAPs and the outcomes include safety, immunogenicity and tolerability.
Product registration path	Following licensure by a WHO listed authority, MR–MAPs should be eligible for prequalification by WHO; and should comply with its programmatic suitability for prequalification guidelines.	The registration path not yet established.	Planned engagement with EMA (EU-M4 all), WHO PQ, NRAs.
MAP components (commercial product)	MAP delivery may need a single-use applicator (while maintaining compliance with packaging requirements). Any patient-contact surfaces of an applicator should be disposable to prevent cross-contamination among vaccinees. The design should include at least one functional, auditory or visual cue as an indicator of successful MAP application.	The device consists of a HD-MAP made from medical grade polymer with >1,000 projections coated with vaccine. This is housed in the primary container which is also the integrated applicator. There are no additional components. The device is single-use and auto-disabling, with audible feedback to confirm delivery. The bottom of the device is covered with a peel-off foil to protect HD-MAPs and indicate use.	The MAP does not have an applicator. There is a force feedback indicator providing visual, tactile and audio cues to confirm successful MAP application.

The dissolving MR-MAP candidate is being evaluated in a phase I/II, single-centre, double-blind, double dummy, randomized age de-escalation trial, to assess the safety, tolerability, and immunogenicity in adults, toddlers, and infants in the Gambia (NCT04394689). The coated MAP is being evaluated in a single centre, placebo controlled, partially blind, randomized phase I trial to examine the safety and tolerability of different doses of MR delivered by MAP in healthy young adults, aged 18–50 years, in Australia (ACTRN12621000820808). Both studies will compare responses to subcutaneous MR immunization with N&S (standard of care) after a single vaccination, and will be evaluated by assessing measles and rubella specific IgG and virus neutralizing antibody titres.

Both candidates will proceed to phase II, contingent on acceptable safety and immunogenicity data from phase I studies. These phase II trials are anticipated to start in 2023, as randomized controlled safety and immunogenicity studies in the target population of healthy infants (~9–10 months of age) living in low-income countries, to coincide with visit five of the recommended WHO vaccination schedule. Data for the dissolving MR-MAP in infants will be available from the Gambian study; but age de-escalation in infants for the coated MAP will need to be generated before the initiation of the phase II study. It is anticipated that demonstration of PoC in these target populations will facilitate both MAP platforms to advance, either with MR or other antigens. The phase II success criteria will include safety and reactogenicity in the target population (minimum sample size 150 per MAP format) and relative MR immunogenicity, compared to standard of care. The two-dose MR schedule will be evaluated in phase III trials.

It is assumed that MR-MAP licensure will be based on demonstration of immunological non-inferiority of the MR-MAP compared to the standard of care, requiring safety data with a minimum sample size of 3,000 for MR-MAP in the target population (11). The phase III study will require produced clinical trial material that is representative of the final manufacturing process (assumed fully automated, compliant with current good manufacturing practices).

## Section 3: MR-MAP Manufacturing Considerations

Manufacturing scale up of a novel technology typically involves building an automated pilot line for Phase III (i.e., 1/5–1/10th of expected commercial scale). For MAPs this means a capacity of millions of MAPs annually, followed by a commercial line to support commercialization, producing up to hundreds of millions of MAPs annually. The need to manufacture MAPs under current good manufacturing practices conditions is a barrier to commercialization due to the significant financial investment and time to build novel manufacturing equipment and a production facility. Dissolving and coated MAPs cannot be produced on the same manufacturing line and thus necessitates separate investment to develop the initial and full-scale manufacturing facilities. While pre-MAP production activities may be similar across the platforms, the exact formulation will depend on the type of MAP and antigen.

Given that the demand and willingness-to-pay for MR-MAPs is currently unknown (see section 7 The need to define the market and articulate the value proposition for MR-MAPs), the proposed strategy is to license and initiate implementation from the pilot scale to enable accelerated access to MR-MAPs by countries and/or procurement organizations. A MAP pilot line could be built and qualified in 2–3 years for ~USD 20–40M (personal communications with MAP developers). This pilot manufacturing facility can be used to support early supply and then be expanded to a commercial full-scale manufacturing facility using modular and flexible approaches when the demand to justify investment is sufficient (12, 13).

Significant concentration of the bulk antigen is required to achieve dry formulation on a MAP and to ensure delivery of a full dose. There may be substantial antigen loss incurred during the concentration and drying process (12, 13). Understanding cost implications of the antigen volume needed for a MAP presentation compared with vials will be important, as the cost of the antigen may be a significant proportion of the total MR-MAP cost. Flexibility of the pilot line to allow manufacture of other MR-related MAPs (e.g., measles mumps rubella) or other vaccine or drug MAPs would enable cost sharing and reducing the risk of the investment. If feasible, MAP manufacturing lines should be adaptable to vaccines or essential medicines with time and budget allotted for change-over between campaigns; however, some active pharmaceutical ingredients such as hormonal contraceptives may require dedicated facilities due to containment risks. While additional regulatory or manufacturing challenges would be expected with this multi-product facility approach, the anticipated benefits of cost sharing and reducing the risk of the investment could facilitate development of MAPs as a platform (12, 13).

The sterility requirements for regulatory approval remain uncertain. End filtration, as used for most vaccines during fill and finish (14), is not possible for MR bulk vaccine due to the size of the measles virus and the high viscosity of concentrated MR formulations (13, 14). The MR-MAP cannot be terminally sterilized since MR antigens are sensitive to heat, radiation, and other methods of sterilization. For this reason, the MAP developers are designing low bioburden or aseptic MAP manufacturing processes. The upfront investment required and costs to acquire aseptic/sterile input materials and to control an aseptic process will be higher compared to a low bioburden process. However, the decrease in manual manipulation and lower risk of lot failure may eventually recuperate those sunk costs.

## Section 4: Considerations Pertinent to MR Antigen Supply and Their Impact on Trials and Timelines

The phase I MR-MAP clinical studies are being performed with MR bulk antigen provided by Serum Institute of India Pvt. Ltd (SIIPL), and there is a commitment from SIIPL to provide antigen for the phase II clinical studies. SIIPL's vaccine is one of only two WHO pre-qualified MR vaccines (15) currently, both of which are live, bivalent, attenuated virus vaccines, produced in well-established stationary culture systems. The limited number of prequalified MR vaccine manufacturers for partnership presents an investment risk for MAP technology developers and funders, who require a line of sight and commitment to commercialization of the MR-MAP product. For this reason, there are efforts to encourage vaccine manufacturers of other measles-containing vaccines to produce and prequalify MR antigens. BMGF is currently funding Univercells and Batavia Biosciences to develop a novel MR manufacturing process to enable sustainable, affordable supply, by minimizing equipment and facility related capital investment and lowering operating costs (16).

While a new source of antigen potentially mitigates the partnership and commercialization risk for MR-MAP development, it presents other complexities. Switching to an MR antigen that is manufactured in a different facility or by a different manufacturer may require formulation optimization to enable full dosing and improved thermostability of the MAP format. Such significant change to the MAP will likely require a clinical bridging study to demonstrate safety and immunological non-inferiority between the two antigen sources in the target population, before it is approved. If a “switch” from the current SIIPL vaccine happens late in product development (i.e., between phase II and III, or during phase III), it will delay the implementation of MR-MAPs by 2–5 years. Therefore, if there is a need to change the source or formulation of the MR antigen, these changes will need to be made as soon as possible.

## Section 5: Projected Timelines for MR-MAP Clinical Development, Manufacturing, Licensure and WHO Prequalification

**Figure 1:**
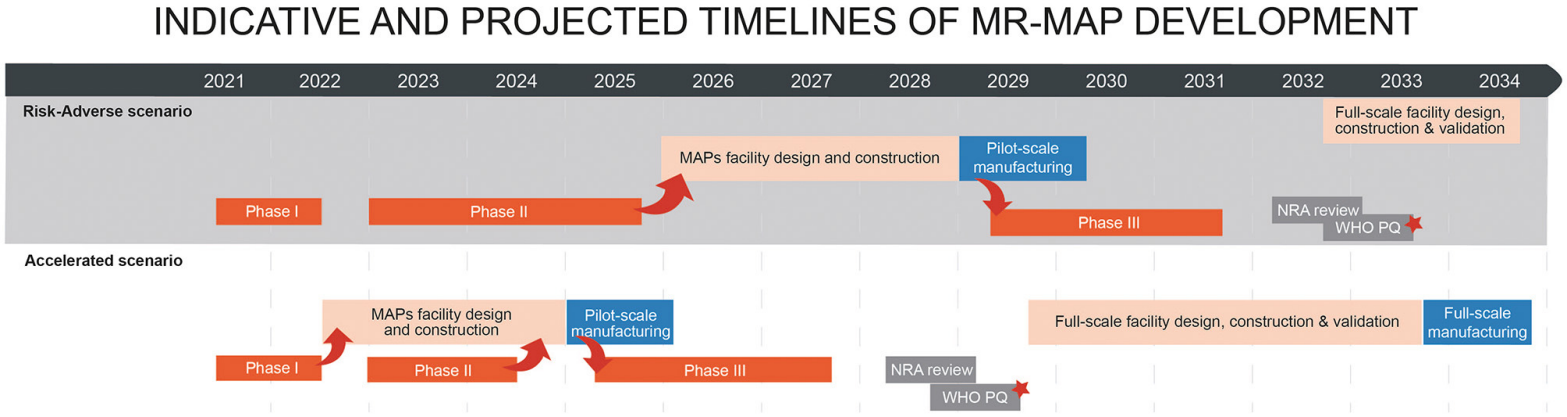
Alternative timelines for MR-MAP development from phase one trial to WHO prequalification and product launch. Arrows indicate flow of data/materials between clinical and manufacturing activities. The timelines do not reflect a timeline for any MR-MAP product. The actual timelines may vary. *MR-MAP is ready to be used in LMICs. See assumptions behind the scenarios in Section 5.

Broadly speaking, there are two scenarios to achieve MR-MAP WHO prequalification, with different risk profiles, timelines and associated assumptions (Figure 1).

The current, “risk-adverse” scenario assumes funding the manufacturing scale and late-stage clinical development of only one of the two MR-MAP candidates currently in phase I clinical studies. This requires a down selection on the basis of PoC data from the phase II clinical study, and will be informed by other factors such as thermostability, human factors, manufacturability and cost of goods. This assessment will be a pre-requisite for investment in the pilot MAP manufacturing line and to initiate the phase III trial. As such, the length of the phase II trial for the risk-adverse scenario in Figure 1 reflects the total time required to complete phase II trials, as well as the broader data assessment for both MR-MAP candidates. There will likely be an additional delay after selection of the optimal MR-MAP format to establish commercial terms, funding and partnerships, including between MAP developer and vaccine manufacturers. As such, the phase III trial could start in 2029 with WHO prequalification by 2033, at the earliest under this scenario.

The alternative “accelerated” scenario does not require a selection of a single MR-MAP candidate at the end of the phase II trial. It assumes at-risk investments in the design, construction, and validation of a pilot manufacturing facility during the phase I and II trials, to position the production of MR-MAPs for phase III immediately following clinical PoC. This would enable the phase III trial to start as early as 2025, and WHO prequalification by 2029. The timeline for phase II is shorter than in the risk-adverse scenario as it is completed independently of the “other” MR-MAP candidate and the criteria for initiating preparations for phase III do not necessarily include data on thermostability, human factors, cost of goods, and manufacturability assessments.

In both scenarios, the MR-MAP is licensed at pilot scale, and the design, construction, and validation of the full-scale manufacturing facility would start after WHO PQ, driven by the market. Both scenarios assume ambitious targets for transition between trials, highlighting the urgency for meticulous planning and alignment of activities and stakeholders. The scenarios also assume committed antigen supply for all phases of clinical development and no antigen “switch” during development.

The COVID-19 pandemic has fundamentally reshaped expectations with regards to the speed of vaccine development, in general. The momentum of COVID-19 vaccine development is likely to continue, and investment in COVID-MAP development may accelerate MR-MAPs. However, the funding, market, and manufacturing environments are drastically different for COVID-19 vaccines and MR. COVID-19 is a disease with significant burden in high-income countries, creating an attractive market for vaccine manufacturers. The rapid development of COVID-19 vaccines resulted from intensified funding, political imperatives and co-ordinated effort from vaccine stakeholders. Lastly, the manufacturing of COVID-19 vaccines leveraged existing manufacturing facilities, whereas manufacturing of MR-MAPs requires significant investment in novel production facilities.

### Assumptions—Common to Both Scenarios

- *Timelines assume no switch of MR vaccine antigen. If a switch occurs, it will likely require a clinical bridging study that will delay licensure*.
- *Pilot-scale manufacturing is required to produce material for the phase III study. Further scale-up will be performed post-licensure via variations to the license application*.
- *The phase III study timeline is currently an estimate; the study has not been designed and there are currently no statistically robust sample size/timeline calculations*.
- *One year required for the National Regulatory Authority (NRA) review, that could be in parallel to PQ, particularly through the EMA M4 all process*.
- *Licensure, PQ and commercial launch is from the pilot MAP manufacturing facility; the MR-MAP manufacturer may use modular and flexible approaches to scale up the production to meet the demand*.
- *The timing of establishing the full-scale facility will depend on demand. The timeline does not account for demand forecasts, and thus the timing of the full-scale facility may shift from how it is depicted*.
- *Assumes funding is put in place, partners and pilot facility site are identified, and commercial terms are agreed after Phase II PoC*.

### Risk-Adverse Scenario

*Note: This strategy enables a comparison of both MR-MAP formats at the completion of phase II, across multiple factors, to inform a decision to **advance one of the two** MR-MAPs to phase III*.

- *Phase II will be a clinical PoC to evaluate immunogenicity of a single dose of each MR-MAP formats, relative to standard of care in 9–10-month-old children, in LMICs. Candidates that meet their clinical endpoints, as well as other “success” criteria such as thermostability, human factor, cost of goods and manufacturability assessments will proceed to phase III*.
- *A decision to invest in a pilot facility is contingent on receiving full data packages for both MR-MAP candidates, post phase II. The timeline of the phase II trial in the baseline scenario reflects the time needed to complete the phase II trial for both MR-MAP candidates, but assumes partners and pilot facility site are identified, and commercial terms are agreed during phase II studies*.
- *Investment in building pilot facility **after** phase II PoC data and candidate down-selection. Additional data demonstrating that an MR-MAP meets the minimum requirements of the WHO Target Product Profile may be requested*.
- *Three years for MAP facility design, construction, and validation*.

### Accelerated Scenario

- *The timeline for phase II is 18 months, as it is completed independently of the “other” MR-MAP candidate*.
- *The success criteria for initiating phase III include immunogenicity, safety and reactogenicity, but the expectation for thermostability, human factors, cost of goods, and manufacturability assessments may be different then for the risk-adverse scenario*.
- *Investment in building the initial manufacturing facility **before** phase II PoC data. Assumes funding is in place, partners and facility site are identified, and commercial terms are agreed during phase I studies*.
- *Assumes 2.5 years required for MAP facility design, construction and validation data packages required for investment may vary from the risk adverse scenario*.

## Section 6: Clarity and Considerations for the MR-MAP Regulatory Strategy

The manufacturing process together with factors such as the vaccine source; the location of MAP production; and the license-holder of the MR-MAP end product (vaccine manufacturer or MAP developer) will inevitably impact the regulatory pathway for MR-MAPs. For example, the MR vaccine might be produced in a different country than the production of the MR-MAPs, and thus not registered in the latter. Determination of the likely NRA that will issue a market authorization license for the MR-MAP is important to be able to obtain early feedback on regulatory expectations and will inform the clinical strategy. For example, some NRAs such as the Drug Controller General of India require clinical studies to be conducted domestically. Until these factors are finalized, it is challenging to chart a regulatory strategy for the product.

An MR-MAP would be a combination product (vaccine and device), with the primary mode of action being its vaccine (biologic) component. NRAs have different approaches to reviewing combination products, but device-related expectations include adherence to design controls and provision of human factors data. As a relatively new technology class, several Critical Quality Attributes for MAPs are unique and will require novel test methods to be developed and justified to regulators. Through PATH's MAP Center of Excellence, a Regulatory Working Group has been established, composed of representatives from academic and commercial MAP developers, vaccine manufacturers, NRAs, pharmacopeia, and WHO PQ, to provide recommendations on Critical Quality Attributes for the MAP technology class and how they can be demonstrated through standardized tests. PATH and WHO have begun to engage with the African Vaccine Regulatory Forum (17) and the African Medicines Devices Forum (18) on vaccine-MAP products and to initiate discussions related to the MR-MAP clinical development plan and approval pathway, which may include the European Medicine Agency Medicines for use outside the EU procedure (M4 all) (19) to accelerate and facilitate WHO prequalification.

## Section 7: The Need to Define the Market and Articulate the Value Proposition for MR-MAPs

Immunization stakeholders are aligned on the public health need for MR-MAPs. However, MR-MAPs will cost more than the current N&S presentation. The higher price per dose of MR-MAPs is expected to be off-set by savings in reduced vaccine wastage, logistics and service delivery costs to immunization programs and by broader health and economic gains from improved and equitable MR coverage. However, these trade-offs need to be quantified to generate demand from countries and interest from funders, and to support discussions on willingness-to-pay. In addition, vaccine manufacturers and MAP developers will need to understand the market opportunity and business case to inform their investment decisions. As such, the “value” of MR-MAPs from the perspective of different stakeholders needs to be assessed to incentivise sustained product development and eventual uptake.

The value and potential impact of MR-MAPs depends on critical product attributes, such as efficacy, simplified administration, thermostability and packed volume, as described in the Target Product Profile (20). Few studies have evaluated the value proposition for MAPs (21–23) and only one has focused on MR-MAPs (22). The latter study assumed no price premium for MR-MAPs and found that an MR-MAP could bring programmatic savings of $0.70 per dose because of savings on cold chain storage, injection equipment, administration time, sharps waste disposal, and wastage—components of total systems effectiveness (20, 24).

UNICEF together with MM Global Health and the London School of Hygiene and Tropical Medicine are developing an *initial* full vaccine value assessment of MR-MAPs from a public health and societal perspective, accounting for financial and economic costs, to provide input into an investment case (25). It is anticipated that the *initial* full vaccine value assessment will serve as an early foundation to inform decision-making but will require subsequent revisions as additional evidence about MAP attributes, health impact, and cost becomes available. The methodology developed may be used to assess full value of other vaccines delivered via MAPs, informing a MAP “portfolio approach” that may help pave the way (26) for future introduction of vaccine-MAPs in vaccination programs.

**Figure 2:**
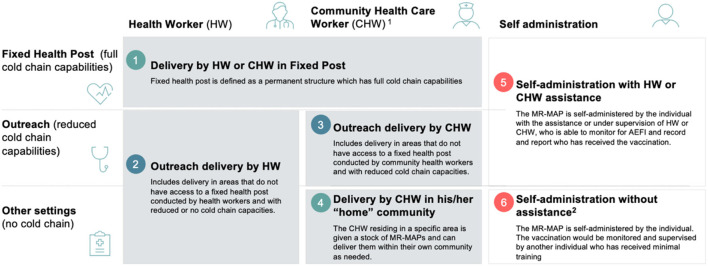
Six use cases for MR-MAPs by service provider (top X axis) and service location (left Y axis). ^1^ Community health worker provide health education, referral and follow-up, case management and basic preventive health care and home visiting services to specific communities. They provide support and assistance to individuals and families in navigating the health and social services system. Occupations included in this category normally require formal or informal training and supervision recognized by the health and social services authorities. ^2^ This may include community member assistance (e.g., teachers, elders, etc.) who have not been trained in MAPs but can monitor and document the administration.

From a vaccine manufacturer and MAP developer perspective, the certainty of the MR-MAP market is key to defining the business case. High-level demand forecast analyses can inform manufacturers of the investment required to manufacture MR-MAPs at the appropriate scale, allowing them to estimate the related cost of goods. Given the expected price premium for MR-MAPs, it is assumed that MR-MAPs will be deployed in specific use case scenarios, such as outreach or outbreak, alongside N&S MR for routine immunization. WHO, together with MM Global Health and several country/regional level stakeholders, identified six use cases for MR-MAP that vary by service delivery location or service provider (Figure 2). *Delivery during outreach by community health worker* (use case 3), *Delivery by community health worker in “home” community* (use case 3), and *Delivery during outreach by trained health worker* (use case 2) are identified as use cases where MR-MAPs could most effectively increase reach and equity The analysis suggests that MR-MAPs will be delivered together with MR N/S within the same immunization programme, however, the programmatic feasibility and acceptability of such approach is unknown. This critical question must be addressed through implementation research during product development, to avoid a delay in implementation once the product is licensed.

WHO and the U.S. Centers for Disease Control and Prevention are also conducting analyses to understand the demand forecast for MR-MAPs for the six use cases. Such analyses need to be dynamic as there are many factors that can influence the total market potential for MR-MAPs in the coming years, such as shifts in the epidemiology of MR, changes in immunization services, and development of the available reimbursement and (co-financing mechanisms for countries).

The market opportunity for MR-MAPs for use in LMICs was recently evaluated by PATH and Linksbridge SPC (27). The analysis found that out of the compared scenarios, the strategy of targeting hard-to-reach populations would generate the highest return on MR-MAP investment from the perspective of a MAP developer. The analysis highlights the need for a combination of innovative market shaping strategies including demand guarantees, commitments on willingness to pay/acceptable price premiums compared to per-dose prices for multidose vials, and direct financial support for the manufacturing and development process to increase manufacturer revenues and improve the attractiveness of the investment.

## Section 8: The Need for Stakeholder Alignment and Integrated Strategic Planning to Advance MR-MAP to Impact

Alignment and coordination across the multitude of stakeholders involved in driving the development, licensure, and uptake of MR-MAPs will be fundamental to the success of this initiative (28). In 2020, the Gavi-led Vaccine Innovation Prioritization Strategy (VIPS) Alliance (including WHO, UNICEF, BMGF and PATH) identified MAPs as the highest priority innovation to overcome the most significant barriers to vaccine delivery, with the potential to increase equitable coverage and contribute to pandemic preparedness. This aligned position was broadly communicated to inform future investment decisions. The VIPS Alliance has since developed an integrated end-to-end (from product development to country uptake) strategy for MAPs, with the input of various immunization partners, including vaccine manufacturers and MAP developers, to accelerate the development and future country uptake of MAPs in LMICs (26).

With a focus on creating and sustaining investment to advance the MAP platform for LMIC use, the VIPS strategy includes prioritizing vaccines for development with MAPs (i.e., the “platform view”), clarifying potential use cases and demand, understanding the willingness to pay based on anticipated health and economic impact, identifying research gaps and implementation/policy related questions, and defining investment cases.

The accelerated development of MR-MAPs is a clear priority of VIPS, who is now seeking synergies with other MAP stakeholders including those advancing the platform for COVID-19 vaccines and pandemic preparedness and response. VIPS acknowledges that in addition to push funding for early product development, a novel pull strategy will be important to ensure incentives to undertake late-stage MR-MAP development and commercialization by vaccine manufacturers. Such pull mechanisms may include quantifying and communicating a potential demand forecast or establishing appropriate procurement and financing mechanisms.

## Section 9: Summary, Conclusions and Recommendations

Despite impressive gains in child survival, quality of life and disability prevention due to MR vaccination, stagnating of global coverage during the past decade resulted in a marked worldwide resurgence of measles in 2018–2019. The deleterious impacts of the COVID-19 pandemic on routine immunization coverage, mass vaccination campaigns to address immunity gaps, and MR surveillance have led to ominous forecasts of a massive pending global measles resurgence (29). The unprecedented infectivity of measles virus makes it the perfect marker for monitoring progress toward achieving universal healthcare coverage, as it will reveal immunity gaps wherever they exist. Thus, although MR eradication is considered biologically and technically feasible, to address inequitable access to child health services and eradicate measles in practice, new tools are required so that hard-to-reach communities have guaranteed access to MR vaccine protection.

Microarray patches that deliver MR vaccine reliably have considerable advantages over N&S delivery. However, without immediate large-scale investment in pilot-scale manufacturing infrastructure, MR-MAPs will not be prequalified by the WHO before 2033 at the earliest. An accelerated introduction scenario is possible, but it would require this investment at-risk, i.e., in parallel to the phase I and II studies. Without a commercial incentive in the form of clear demand and financing strategy, vaccine manufacturers will be reluctant to invest in MR-MAP development, extending the timeframe to licensure and access. Flexibility of the pilot manufacturing line to allow manufacture of other vaccine-MAPs is likely to be an additional important consideration for vaccine manufacturers' decision-making.

Although the scientific and public health community are unanimous about the potential game-changing nature of MR-MAPs to eradicate measles, the unit cost of MR-MAPs will be higher than the currently available MR vaccines. This may be off-set in part by savings in programmatic delivery costs, as well as health and economic gains, however the full value for recipient countries and communities—necessary to drive demand and entice manufacturing investment—needs to be better quantified and articulated. In addition, the feasibility of including an MR-MAP presentation, alongside N&S delivered vaccines within the same immunization programme needs to be addressed.

The work of VIPS in facilitating the assessment of the full value of vaccine-MAPs from the perspective of multiple stakeholders, including country immunization stakeholders and policy makers; vaccine manufacturers; MAP developers; and funders and procurers, as well as of the pull incentives required, could not be more timely or critical (30).

Additional challenges must also be overcome. Having only two prequalified MR vaccine manufacturers is a great concern, but a switch to a new antigen supplier during the MR-MAP development would further delay market introduction of MR-MAPs, especially if this occurs late in product development. The safety profile of the existing MR vaccines is outstanding, and formulation of a new MR vaccine would likely require immunological bridging and safety studies further delaying time to market. Furthermore, combination vaccine-device products pose regulatory questions, and clarity on critical quality attributes is urgently needed.

With current vaccine presentations we are unable to close measles coverage gaps and ensure that measles and rubella are eradicated. Funders and procurement decision makers will need to acknowledge that more expensive presentations are required to achieve these goals. MR-MAPs are poised to contribute to realizing the dream of measles eradication and reach the ~20 million children in every annual birth cohort that are currently missing out on life-saving MR vaccines. This is a critical opportunity for global public health that cannot afford to be missed.

## Author Contributions

All authors listed have made a substantial, direct, and intellectual contribution to the work and approved it for publication.

## Funding

This work was funded by BMGF funding to WHO (INV-005318).

## Author Disclaimer

The authors alone are responsible for the views expressed in this article and they do not necessarily represent the views, decisions or policies of the institutions with which they are affiliated.

## Conflict of Interest

The authors declare that the research was conducted in the absence of any commercial or financial relationships that could be construed as a potential conflict of interest. The handling editor declared a shared affiliation with one of the author MH-A at the time of review.

## Publisher's Note

All claims expressed in this article are solely those of the authors and do not necessarily represent those of their affiliated organizations, or those of the publisher, the editors and the reviewers. Any product that may be evaluated in this article, or claim that may be made by its manufacturer, is not guaranteed or endorsed by the publisher.
